# Alcohol consumption may be a risk factor for cerebrovascular stenosis in acute ischemic stroke and transient ischemic attack

**DOI:** 10.1186/s12883-024-03627-x

**Published:** 2024-04-23

**Authors:** Yiti Liu, Shuo Gu, Maoyuan Gou, Xiaoyan Guo

**Affiliations:** Department of Neurology, the Affiliated Hospital of Southwest Medical University, Taiping Street, Jiangyang District, Luzhou, 646000 China

**Keywords:** Alcohol, Ischemic stroke, Cerebrovascular stenosis, Atherosclerosis

## Abstract

**Background:**

Atherosclerosis are well established risk factors for ischemic stroke, however the association between alcohol consumption and atherosclerosis is controversial. This study aims to explore the potential correlation between alcohol consumption and cerebral stenosis in patients with acute ischemic stroke and transient ischemic attack (TIA).

**Methods:**

Nine hundreds and eighty-eight patients with first acute ischemic stroke attack or TIA were recruited retrospectively. Alcohol consumption was classified into five consumption categories (non-drinkers, occasional drinkers, < 140 g per week [mild drinkers], 140–279 g per week [moderate drinkers], ≥ 280 g per week [heavy drinkers]). Computed tomography angiography (CTA) and digital subtraction angiography (DSA) were utilized to assess the carotid and cerebral artery in all patients. Five-step scale for degree of stenosis was applied: normal (0, 0 points), mild (< 50%, 1 point), moderate (50–69%, 2 points), severe (70–99%, 3 points), and occlusion (100%, 4 points).

**Results:**

The carotid and cerebral artery stenosis scores were positively correlated with moderate alcohol consumption (B = 1.695, *P* < 0.001). Compared with nondrinkers, moderate alcohol consumption had significant increasing risk of moderate carotid and cerebral artery stenosis (OR = 4.28, 95% CI: 1.47–12.49, *P* = 0.008) and severe stenosis (OR = 4.24, 95% CI: 1.55–11.64, *P* = 0.005) and occlusion (OR = 3.87, 95% CI: 1.65–9.06, *P* = 0.002). Compared with nondrinkers, heavy alcohol consumption patients had significant higher risk of carotid and cerebral artery occlusion (OR = 2.71, 95% CI: 1.36–5.41, *P* = 0.005).

**Conclusions:**

Higher alcohol consumption may associate with higher risk and more severity of carotid and cerebrovascular stenosis.

## Introduction

Stroke was the third leading cause of death and disability worldwide [1]. The incidence of ischemic stroke is increasing every year, with around two-thirds of new stroke cases were ischemic stroke [1]. Several factors, including hypertension, diabetes, hyperlipidemia, heart disease, obesity, smoking, gout, aging, inadequate activity, mental factors, and environmental pollution were established risk factors for ischemic stroke [1–3]. However, the correlation of alcohol and ischemic stroke was debated.

Millwood et al. found a U-shaped association between alcohol consumption and the incidence of ischemic stroke in conventional epidemiology [4], another study showed a J-shaped curve between ischemic stroke and alcohol consumption [5], which means a higher risk of ischemic stroke in non-alcohol and heavy alcohol consumption groups, however a protective effect of mild-moderate alcohol consumption on ischemic stroke. However, a prospective study has shown that even small amounts of alcohol consumption can increase the risk of stroke [6]. Atherosclerosis was the most main cause of ischemic stroke [7, 8]. The relationship between alcohol consumption and carotid-cerebral atherosclerosis remains unclear. Some studies indicated a U-shaped relationship between alcohol consumption and carotid atherosclerosis [9–11], or a J-shaped correlation between alcohol intake and carotid atherosclerosis [12–15]. All above studies showed that non-alcohol consumption and heavy alcohol consumption groups may have higher risk but moderate alcohol consumption population may have lower risk of atherosclerosis. Several studies have described a linear relationship between alcohol consumption and atherosclerosis, with an increasing risk as alcohol consumption increases [16, 17], however there were also evidences which supported only moderate or heavy and sustained alcohol consumption was correlated with carotid atherosclerosis [18, 19].

The diagnostic of atherosclerosis was mainly based on carotid artery ultrasound in former published research. And the participants in most studies were healthy population or population with coronary disease or ischemic stroke. Although there were some studies focus on carotid artery atherosclerosis which was assessed only by carotid artery ultrasound. However, there was no study focus on the effect of different alcohol consumption on severity and distribution of carotid and cerebrovascular atherosclerosis and stenosis assessed by head and neck Computed tomography angiography (CTA) or digital subtraction angiography (DSA). This study intends to research the relationship between pre-stroke alcohol consumption and the occurrence, severity and distribution of carotid and cerebrovascular stenosis in patients with first acute ischemic stroke attack and transient ischemic attack (TIA).

## Materials and methods

Our cross-sectional and observational study included 988 patients (941 AIS and 47 TIA patients) who admitted to the Department of Neurology, Southwest Medical University with the first attack of ischemic stroke between January 2020 and February 2023.

Inclusion criteria: (1) Patients fully met the diagnostic criteria for cerebral infarction as laid out in the Chinese Acute Ischemic Stroke Diagnosis and Treatment Guideline 2018 [20] or met the diagnostic criteria for Stroke and TIA [21]. (2) First-onset of ischemic stroke and onset within 48 h on admission. (3) Trial of ORG 10,172 in Acute Stroke Treatment (TOAST) classification of large-artery atherosclerosis or small-vessel occlusion. (4) Brain computed tomography and magnetic resonance imaging was performed on admission. (5) Age ≥ 45 years. 6). Electrocardiogram, cardiac ultrasound, carotid and cerebral CTA or DSA were examined.

Exclusion criteria: (1) Missing data on age, alcohol consumption, smoking, date of onset and admission. (2) Previous history of stroke. (3) Patients with cardioembolic, other cause, and undetermined cause ischemic stroke with centralized evaluation of TOAST classification. (4) Hematological disease, cancer, brain arteriovenous malformation, autoimmune disease, severe hepatic and renal failure. (5) History of neurological or psychiatric disorders.

Current smokers were defined as individuals who have smoked continuously for more than one year and at least one cigarette in the past six months. Former smokers were defined as participants who had smoking history of at least one year of continuous smoking, however had not smoked even a single cigarette in the past six months. Non-smokers were defined as never smoked or who have smoked occasionally or continuously for less than one year.

Alcohol consumption was collected through inquiring the patients or their families about their drinking habits, including the type, frequency, amount, and duration of alcohol consumption. The alcohol concentration was adjusted based on the following criteria: for white wine, the discount rate is 53%, for beer it is 4%, for rice or yellow wine it is 15%, grape wine 12% [22]. Drinking categories were defined as (a) non-drinkers (less than 280 g per year or drinking continues less than 6 months). If drinking continues for more than 6 months, were grouped into five consumption categories: (b) former drinkers (alcohol consumption before and no drinking in the last 6 months); (c) occasional drinkers (less than 1 times per week and < 140 g per week); (d) mild drinkers (at least 1 time per week and < 140 g per week); (e) moderate drinkers (at least 1 time per week and 140–279 g per week); (f) heavy drinkers (at least 1 time per week and ≥ 280 g per week) [4].

The vessels included the common carotid artery, internal carotid artery, vertebral artery, basilar artery, anterior cerebral artery, middle cerebral artery, and posterior cerebral artery were examined. A five-step scale for degree of stenosis was applied: normal (0, 0 points), mild (< 50%, 1 point), moderate (50–69%, 2 points), severe (70–99%, 3 points), and occlusion (100%, 4 points) [12, 23]. A stenosis score was calculated for each vessel based on the most severe case of stenosis. The total stenosis score was obtained by adding the stenosis scores of all vessels of a patient.

On admission and at discharge, the National Institutes of Health Stroke Scale (NIHSS) [24] and Modified Rankin Scale (mRS) [25] scores were also assessed.

### Statistical analysis

Data were analyzed with SPSS 26.0. All continuous data were presented as mean ± standard deviation, and all categorical variables including sex, pneumonia, gastrointestinal bleeding, smoking history, hypertension, coronary heart disease, medication, and diabetes mellitus were presented as percentages. Vascular stenosis scores were compared using a one-way variance analysis between groups, while the Fisher least significant difference method was used to determine statistical significance. Linear regression model was used to study the association between total intracranial and extracranial artery stenosis scores and each alcohol consumption, after adjusting for age, sex, body mass index (BMI), hypertension, diabetes, coronary heart disease, smoking, triglyceride, and low-density lipoprotein. Odds ratios (OR) are presented with 95% confidence intervals (CIs) and were estimated by using Logistic regression to examine the relationship between different degrees of stenosis in alcohol consumption subgroups (the reference category is non-drinkers), after adjusting for age, sex, body mass index (BMI), hypertension, diabetes, coronary heart disease, smoking, triglyceride, and low-density lipoprotein. A value of *P* < 0.05 was considered statistically significant.

## Results

This study continuously enrolled 988 acute ischemic stroke patients (301 females and 687 males). According to the alcohol consumption, 577 (58.4%) patients were nondrinkers, 151 (15.3%) were occasional drinkers, 37 (3.7%) were mild drinkers, 104 (10.5%) were moderate drinkers, and 119 (12.0%) were heavy drinkers (Table 1). Among drinking patients, 366 (89.1%) patients were current drinkers, while 45 (10.9%) patients were former drinkers (Table 1). Five hundred and ninety-eight (60.5%) patients only received head and neck CTA, and 19 (1.9%) patients only received DSA, while 371 (37.6%) patients had both CTA and DSA examination. We included DSA results when the patient underwent both CTA and DSA, so the diagnosis of cerebrovascular stenosis of 598 patients were based on CTA results, 390 patients were based on DSA results.

**Table 1:** Demographic and clinical characteristics of the included population

	Total Sample		Alcohol Consumption						Drinking Status	
			Non-drinkers	Occasional drinkers	< 140 g/week	140–279 g/week	≥ 280 g/week		Current drinkers	Former drinkers
Number (Female/Male)	988 (301/687)		577 (293/284)	151 (8/143)	37 (0/37)	104 (0/104)	119 (0/119)		366 (8/358)	45 (0/45)
Age	63.2 ± 10.6		64.1 ± 11.0	61.5 ± 10.4	60.9 ± 10.5	63.0 ± 9.5	61.3 ± 9.5		61.4 ± 10.0	65.2 ± 8.6
BMI	24.2 ± 3.3		24.1 ± 3.5	24.6 ± 3.1	24.0 ± 3.5	24.1 ± 2.9	24.0 ± 2.9		24.3 ± 3.0	24.1 ± 2.7
Hypertension	740 (74.9%)		438 (75.9%)	106 (70.2%)	28 (75.7%)	77 (74.0%)	91 (76.5%)		268 (73.2%)	34 (75.6%)
Regular treatment	323 (32.7%)		196 (34.0)	44 (29.1%)	12 (32.4%)	32 (30.8%)	39 (32.8%)		108 (29.5%)	19 (42.2%)
Untreated or irregular treatment	417 (42.2%)		242 (41.9%)	62 (41.1%)	16 (43.2%)	45 (43.3%)	52 (43.7%)		160 (43.7%)	15 (33.3%)
Diabetes	271 (27.4%)		161 (27.9%)	46 (30.5%)	8 (21.6%)	24 (23.1%)	29 (24.4%)		92 (25.1%)	15 (33.3%)
Regular treatment	186 (18.8%)		114 (19.8%)	29 (19.2%)	7 (18.9%)	17 (16.3%)	19 (16.0%)		63 (17.2%)	9 (20.0%)
Untreated or irregular treatment	85 (8.6%)		50 (8.7%)	17 (11.3%)	1 (2.7%)	7 (6.7%)	10 (8.4%)		29 (7.9%)	6 (13.3%)
Coronary heart disease	108 (10.9%)		63 (10.9%)	19 (12.6%)	2 (5.4%)	10 (9.6%)	14 (11.8%)		37 (10.1%)	8 (17.8%)
Regular antiplatelet treatment	21 (2.1%)		15 (2.6%)	4 (2.6%)	1 (2.7%)	0 (0%)	1 (0.8%)		5 (1.4%)	1 (2.2%)
Regular statin treatment	22(2.2%)		15 (2.6%)	4 (2.6%)	1 (2.7%)	0 (0%)	2 (1.7%)		5 (1.4%)	2 (4.4%)
Gastrointestinal bleeding	69 (7.0%)		38 (6.6%)	9 (6.0%)	2 (5.6%)	13 (12.5%)	7 (5.9%)		30 (8.2%)	1 (2.2%)
Pneumonia	221 (22.4%)		114 (19.8%)	39 (25.8%)	9 (24.3%)	29 (27.9%)	30 (25.2%)		96 (26.2%)	11 (24.4%)
Smoking status										
Current smokers	376 (38.1%)		84 (14.6%)	99 (65.6%)	28 (75.7%)	73 (70.2%)	92 (77.3%)		270 (73.8%)	22 (48.9%)
Former smokers	64 (6.5%)		10 (1.7%)	13 (8.6%)	3 (8.1%)	23 (22.1%)	15 (12.6%)		34 (9.3%)	20 (44.4%)
Non-smokers	548 (55.4%)		483 (83.7%)	39 (25.8%)	6 (16.2%)	8 (7.7%)	12 (10.1%)		62 (16.9%)	3 (6.7%)
Leukocyte counts (10^9^/L)	8.7 ± 3.2		8.7 ± 3.4	9.0 ± 2.95	8.1 ± 2.2	8.3 ± 2.7	8.7 ± 3.6		8.7 ± 3.1	8.5 ± 2.7
Neutrophil counts (10^9^/L)	6.5 ± 3.2		6.5 ± 3.3	6.9 ± 3.0	6.1 ± 2.2	6.3 ± 2.6	6.5 ± 3.7		6.5 ± 3.1	6.6 ± 2.6
Lymphocyte counts (10^9^/L)	1.6 ± 0.7		1.6 ± 0.7	1.6 ± 0.8	1.4 ± 0.5	1.4 ± 0.6	1.6 ± 0.6		1.5 ± 0.7	1.4 ± 0.6
Triglyceride	1.8 ± 1.3		1.8 ± 1.3	1.8 ± 1.4	1.9 ± 1.8	1.7 ± 1.1	1.9 ± 1.2		1.8 ± 1.3	1.7 ± 1.3
Low density lipoprotein	3.0 ± 0.9		3.0 ± 0.9	3.1 ± 0.9	3.1 ± 1.1	2.9 ± 0.9	2.8 ± 0.9		3.0 ± 0.9	3.0 ± 1.0
High density lipoprotein	1.3 ± 0.4		1.3 ± 0.4	1.2 ± 0.3	1.2 ± 0.4	1.3 ± 0.4	1.3 ± 0.5		1.2 ± 0.4	1.2 ± 0.3
Creatinine	70.1 ± 19.2		67.1 ± 18.9	73.7 ± 18.2	72.1 ± 18.8	75.0 ± 18.9	75.2 ± 19.4		73.8 ± 18.5	78.9 ± 20.0
Glomerular filtration rate	93.1 ± 20.6		92.1 ± 20.4	95.5 ± 24.2	98.5 ± 24.1	93.1 ± 16.6	93.1 ± 17.9		95.2 ± 21.0	88.7 ± 17.1
Uric acid	339.0 ± 96.4		321.9 ± 92.2	355.7 ± 94.3	387.0 ± 121.1	359.6 ± 88.0	367.9 ± 100.0		362.7 ± 97.8	365.8 ± 92.8
NIHSS score on admission	6.6 ± 5.9		6.5 ± 5.8	6.5 ± 6.8	6.6 ± 5.6	7.4 ± 5.0	6.4 ± 5.7		6.7 ± 6.0	6.7 ± 5.9
NIHSS score at discharge	6.1 ± 8.4		5.8 ± 8.2	6.2 ± 8.3	4.5 ± 3.9	7.2 ± 9.5	6.5 ± 9.5		6.4 ± 8.5	6.2 ± 10.3
mRS score on admission	2.7 ± 1.4		2.6 ± 1.4	2.6 ± 1.4	2.7 ± 1.3	2.9 ± 1.3	2.6 ± 1.5		2.7 ± 1.4	2.6 ± 1.5
mRS score at discharge	2.2 ± 1.6		2.1 ± 1.6	2.2 ± 1.6	2.1 ± 1.3	2.5 ± 1.7	2.2 ± 1.8		2.3 ± 1.6	2.0 ± 1.7

BMI: Body max index, NIHSS: National Institutes of Health Stroke Scale; mRS: Modified Rankin Scale

Table 1 shows the demographic and clinical characteristics of the study population. Non-drinkers (16.3%) had a lower rate of current smoking than occasional (74.2%), mild (83.8%), moderate (92.3%), and heavy (89.9%) drinkers. We did not find significant differences in the prevalence of pneumonia, hypertension and diabetes, triglycerides, low-density lipoprotein, high-density lipoprotein levels, uric acid levels, and glomerular filtration rate among different drinking subgroups (Table 1). There was no significant difference in changes in NIHSS and mRS scores between admission and discharge among different subgroups (Table 1).

**Fig. 1:**
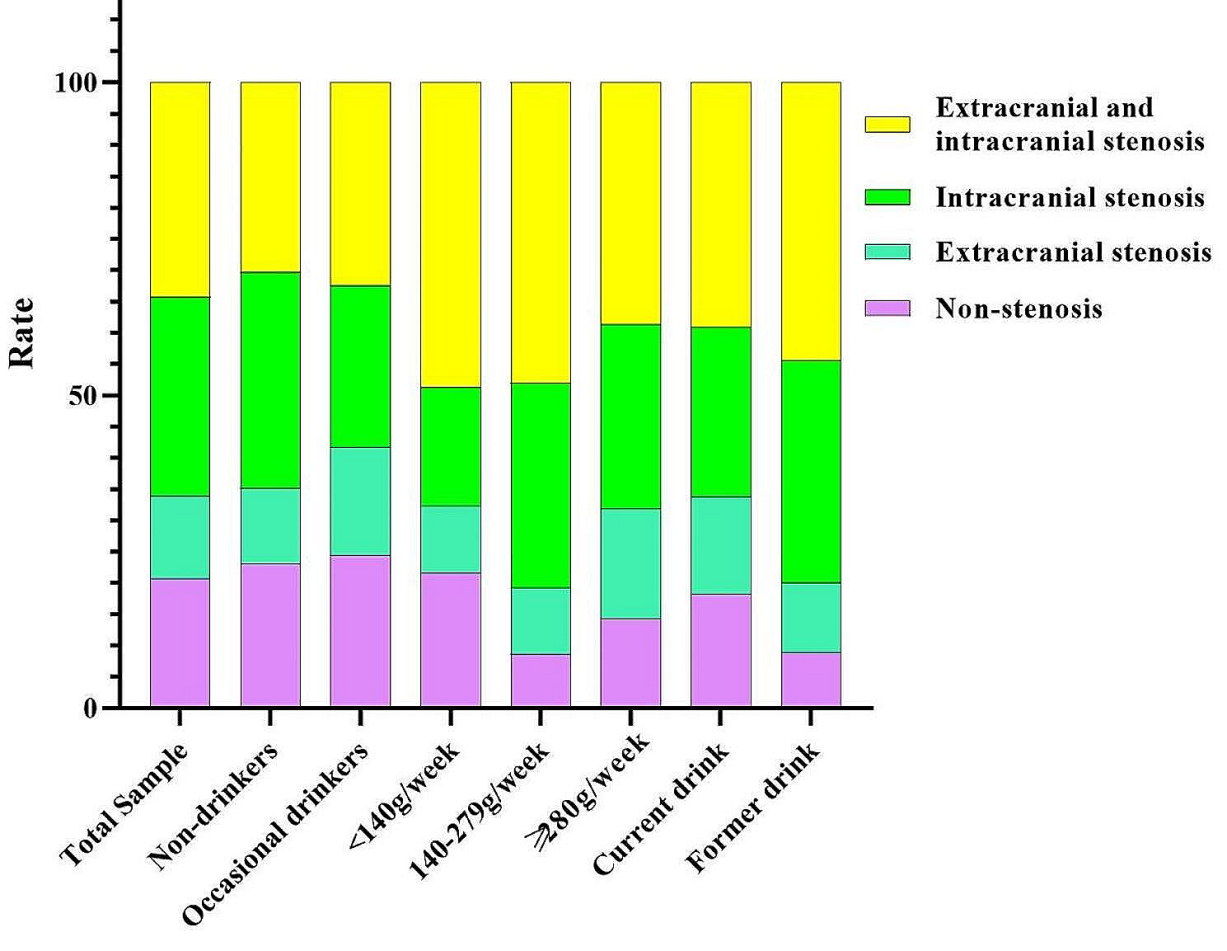
The distribution of intracranial and extracranial vascular stenosis among various subgroups

Among the 988 patients, 205 (20.7%) had no vascular stenosis, 131 (13.3%) had extracranial vascular stenosis, 314 (31.8%) had intracranial vascular stenosis, and 338 (34.2%) had both extracranial and intracranial vascular stenosis (Fig. 1). Moderate drinkers (91.3%), heavy drinkers (85.7%), and former drinkers (91.1%) had a higher incidence of cerebral stenosis than the nondrinking subgroup (79.3%) (Fig. 1). Mild drinkers (48.6%), moderate drinkers (48.1%), heavy drinkers (38.7%), and former drinkers (44.4%) had higher rates of intracranial and extracranial vascular stenosis compared with nondrinkers (30.3%) and occasional drinkers (32.5%) (Fig. 1).

**Fig. 2:**
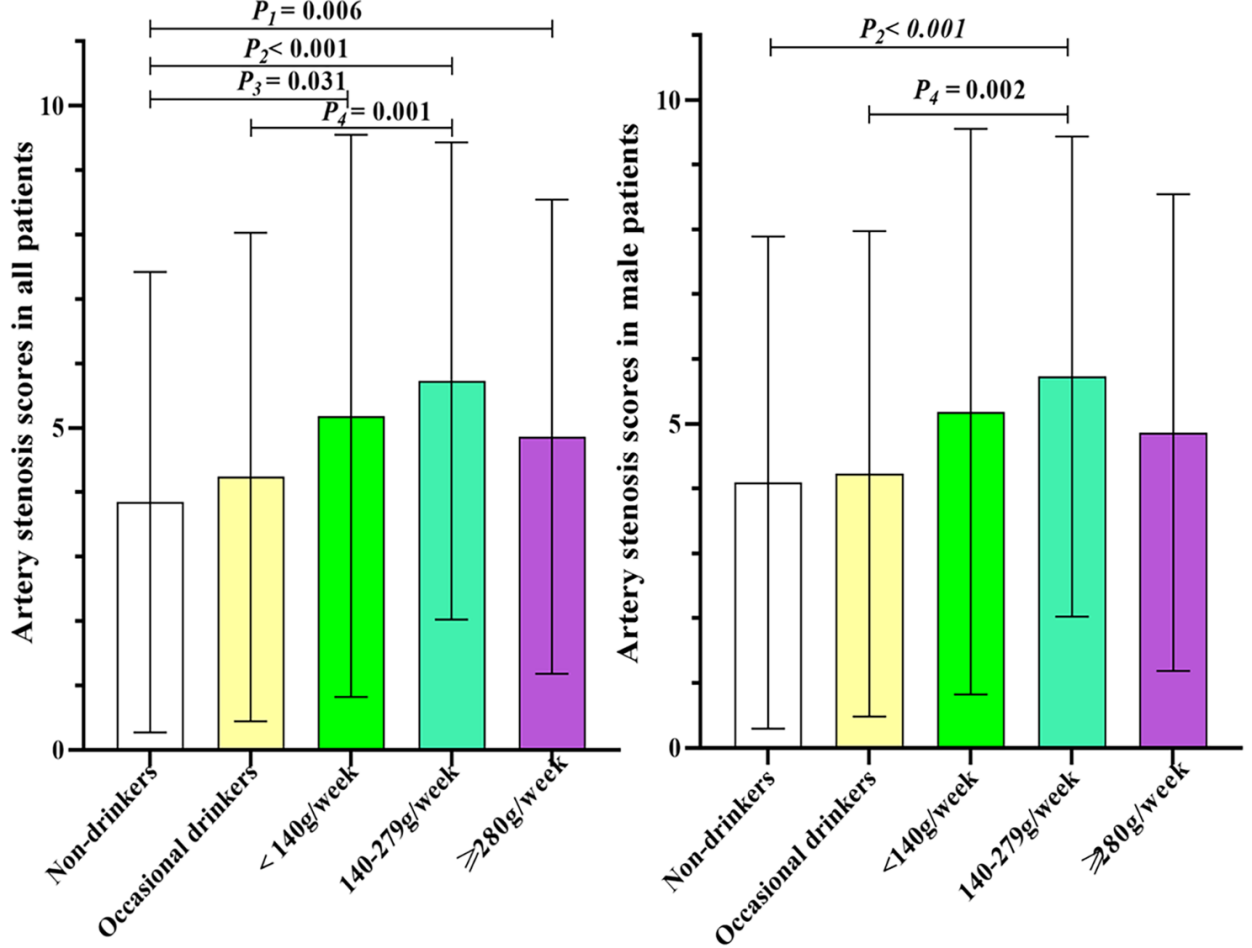
Comparisons of the total intracranial and extracranial artery stenosis scores among subgroups *P*_*1*_: Non-drinkers vs. ≥280 g/week, *P*_*2*_: Non-drinkers vs. 140–279 g/week, *P*_*3*_: Non-drinkers vs. <140 g/week, *P*_*4*_: Occasional drinkers vs. 140–279 g/week

Figure 2 shows the comparisons of the total carotid and cerebral arteries stenosis score in different drinking subgroups. The non-drinking group had the lowest mean carotid and cerebral artery stenosis score, followed by the occasional drinking group, the heavy drinking group, and the light drinking group, whereas the individuals who consumed alcohol moderately had the highest score. The carotid and cerebral artery stenosis scores were higher in the mild, moderate and heavy drinking groups compared to the nondrinking group (5.19 ± 4.36 and 5.73 ± 3.71 and 4.87 ± 3.68 vs. 3.85 ± 3.58; *P* = 0.031, *P* < 0.001, *P* = 0.006, respectively) (Fig. 2). The moderate drinking group also had higher carotid and cerebral artery stenosis scores compared to the occasional drinking group (5.73 ± 3.71 vs. 4.24 ± 3.80, *P* = 0.001) in all patients (Fig. 2). The moderate drinking group also had higher carotid and cerebral artery stenosis scores compared to the non-drinking and occasional drinking group, which were also observed in the male subgroup (Fig. 2).

A multivariable linear regression model was used to estimate the correlation between different levels of alcohol consumption and total intracranial and extracranial artery stenosis scores (Table 2). The carotid and cerebral artery stenosis scores were positively correlated with moderate alcohol consumption (B = 1.695, *P* < 0.001) (Table 2). However, we also found that mild and heavy drinking groups (B = 1.230, *P* = 0.056; B = 0.829, *P* = 0.055, respectively) had positive trend association with higher carotid and cerebral artery stenosis scores, although the results were not statistically significantly different (Table 2). The above correlations were also observed in the male subgroup (Table 2).

**Table 2:** Multivariable predicting model to estimate the correlation between different alcohol consumption and the total intracranial and extracranial artery stenosis scores

Model in all patients^a^	B	SE	β	t-value	*P*-value
Non-drinkers (Reference)					
Occasional drinkers	0.227	0.379	0.022	0.579	0.550
< 140 g/week	1.230	0.642	0.063	1.917	0.056
140–279 g/week	1.695	0.458	0.137	3.620	< 0.001^*^
≥ 280 g/week	0.829	0.431	0.073	1.923	0.055^*^
Model in male patients^b^	B	SE	β	*t-value*	*P-*value
Non-drinkers (Reference)					
Occasional drinkers	0.142	0.415	0.015	0.343	0.732
< 140 g/ week	1.163	0.676	0.069	1.721	0.086
140–279 g/week	1.603	0.486	0.150	3.300	0.001^*^
≥ 280 g/week	0.800	0.457	0.079	1.748	0.081^*^

^a^Adjusted for age, sex, body max index, hypertension (Non-hypertension group, untreated or irregular treatment group, regular treatment group), diabetes (Non-diabetes group, untreated or irregular treatment group, regular treatment group), coronary heart disease, antiplatelet treatment, statin treatment, smoking (Non-smokers, former smokers group, current smokers group), triglyceride, low density lipoprotein. ^b^ Adjusted for age, body max index, hypertension (Non-hypertension group, untreated or irregular treatment group, regular treatment group), diabetes (Non-diabetes group, untreated or irregular treatment group, regular treatment group), coronary heart disease, antiplatelet treatment, statin treatment, smoking (Non-smokers, former smokers group, current smokers group), triglyceride, low density lipoprotein. SE: Standard error; ^*^significant difference

The association between alcohol consumption and the degree of carotid and cerebral artery stenosis is shown in Table 3. Logistic regression shown that moderate alcohol consumption significantly increased the risk of moderate carotid and cerebral artery stenosis (OR = 4.28, 95% CI: 1.47–12.49, *P* = 0.008) and severe stenosis (OR = 4.24, 95% CI: 1.55–11.64, *P* = 0.005) and occlusion (OR = 3.87, 95% CI: 1.65–9.06, *P* = 0.002) compared with nondrinkers (Table 3). Heavy alcohol consumption significantly increased the risk of carotid and cerebral artery occlusion compared with nondrinkers (OR = 2.71, 95% CI: 1.36–5.41, *P* = 0.005) (Table 3). The above differences were also observed in the male subgroup (Table 3).

**Table 3:** Adjusted odds ratios (OR) and 95% confidence intervals (CI) of different Stenosis degrees in each alcohol consumption subgroup

	Mild stenosis			Moderate stenosis			Severe stenosis			Occlusive	
	OR (95% CI)	*P*-value		OR (95% CI)	*P*-value		OR (95% CI)	*P*-value		OR (95% CI)	*P*-value
In all patients^a^											
Non-drinkers (Reference)	1.00			1.00			1.00			1.00	
Occasional drinkers	0.49 (0.25, 96)	0.037^*^		0.75 (0.31, 1.81)	0.518		1.32 (0.66, 2.64)	0.434		1.07 (0.61, 1.87)	0.813
< 140 g/week	0.54 (0.16, 1.87)	0.329		1.05 (0.24, 4.54)	0.951		2.07 (0.64, 6.64)	0.223		1.34 (0.50, 3.56)	0.559
140–279 g/week	1.48 (0.57, 3.89)	0.422		4.28 (1.47, 12.49)	0.008^*^		4.24 (1.55, 11.64)	0.005^*^		3.87 (1.65, 9.06)	0.002^*^
≥ 280 g/week	0.89 (0.39, 2.03)	0.778		1.62 (0.59, 4.44)	0.347		1.75 (0.70, 4.40)	0.233		2.71 (1.36, 5.41)	0.005^*^
In male patients^b^											
Non-drinkers (Reference)	1.00			1.00			1.00			1.00	
Occasional drinkers	0.37 (0.18, 0.75)	0.006^*^		0.81 (0.32, 2.09)	0.666		1.11 (0.53, 2.36)	0.780		0.96 (0.54, 1.73)	0.899
< 140 g/week	0.54 (0.16, 1.85)	0.328		1.20 (0.27, 5.35)	0.814		2.04 (0.62, 6.68)	0.241		1.25 (0.47, 3.35)	0.654
140–279 g/week	1.36 (0.51, 3.59)	0.501		4.60 (1.52, 13.98)	0.007^*^		3.84 (1.37, 10.75)	0.010^*^		3.60 (1.53, 8.50)	0.003^*^
≥ 280 g/week	0.84 (0.36, 1.95)	0.720		1.77 (0.62, 5.07)	0.288		1.63 (0.63, 4.19)	0.310		2.58 (1.28, 5.22)	0.008^*^

^a^Adjusted for age, sex, body max index, hypertension (Non-hypertension group, untreated or irregular treatment group, regular treatment group), diabetes (Non-diabetes group, untreated or irregular treatment group, regular treatment group), coronary heart disease, antiplatelet treatment, statin treatment, smoking (Non-smokers, former smokers group, current smokers group), triglyceride, low density lipoprotein. ^b^ Adjusted for age, body max index, hypertension (Non-hypertension group, untreated or irregular treatment, regular treatment group), diabetes (Non-diabetes group, untreated or irregular treatment group, regular treatment group), coronary heart disease, antiplatelet treatment, statin treatment, smoking (Non-smokers, former smokers group, current smokers group), triglyceride, low density lipoprotein; *significant difference

## Discussion

We found that the carotid and cerebral artery stenosis scores were higher in the moderate and heavy drinking groups compared to the nondrinking group. A multivariable linear regression model indicated that the carotid and cerebral artery stenosis scores were positively correlated with moderate alcohol consumption. Logistic regression shown that moderate alcohol consumption significantly increased the risk of moderate, severe carotid and cerebral artery stenosis and occlusion, compared with nondrinkers. In addition, heavy alcohol consumption significantly increased the risk of carotid and cerebral artery occlusion compared with nondrinkers.

Overall, we found that moderate and heavy alcohol consumption may be associated with more severity of atherosclerosis in carotid and cerebral artery which is consistent with other studies [10, 11, 13–15, 18, 26]. The link between heavy drinking and atherosclerosis has been well established. Atherosclerosis is characterized as a chronic inflammatory disease in which the artery wall thickens due to the accumulation of cholesterol, macrophages, and smooth muscle cells (SMCs), resulting in reduced blood flow through the artery [27]. The precise mechanism of ethanol resulting in atherosclerosis is unclear. However, multiple biochemical and physiological effects may be involved. Firstly, alcohol mediates the change of nitric oxide (NO) which may be an inducing factor of atherosclerosis. The potential mechanisms include the rapid activation of mitochondrial aldehyde dehydrogenase 2, the change of ethanol-induced vasodilator prostacyclin 2 or potent vasoconstrictor endothelin-1, and the change of intracellular Ca^2+^ and Mg^2+^ levels which are all involved in the pathophysiological processes of atherosclerosis [27]. Secondly, high concentrations of alcohol were found to decrease hypersensitive epithelial resistance, increase cell adhesion molecule (CAM) expression, and interleukin-6 production, while increasing monocyte chemotactic protein-1 expression and monocyte adhesion, thereby promoting inflammation and increasing the production of reactive oxygen species (ROS) which play a critical role in atherosclerosis [28]. ROS resulting in lipid peroxidation, protein oxidation, production of proinflammatory cytokines, and activation of mitogen-activated protein kinase (MAPK) and further leading to endothelial dysfunction [29–35]. Additionally, there is extensive evidence support that alcohol consumption is correlated with hypertension [4, 36–38] which may accelerate the genesis of atherosclerosis. Numerous mechanisms, including increased sympathetic nervous system activity, increased intracellular Ca^2+^ levels in vascular smooth muscle cells (VSMCs), increased renin-angiotensin system (RAS) activity, and endothelial dysfunction, had been implicated in the association between heavy alcohol consumption and hypertension [39–41]. However, our study found the moderate and heavy alcohol consumption was associated with more severity of atherosclerosis in carotid and cerebral artery after adjusted other influencing factors such as age, sex, BMI, hypertension, diabetes, coronary heart disease, antiplatelet treatment, statin treatment, smoking, triglyceride, and low-density lipoprotein. So, we speculate that alcohol consumption promotes the development of atherosclerosis may be through endothelial dysfunction more.

The relationship between occasional or mild alcohol consumption and atherosclerosis is still a continuous topic of debate. We did not find any significant correlation between occasional or mild alcohol intake and the atherosclerosis. This is consistent of some studies [18, 42], however it is not agreement with previous researches which indicated that mild alcohol consumption provided a protective effect on carotid-cerebral artery [10, 15, 26]. Various factors may be involved in the discrepancy between our study and other studies. First, mild alcohol consumption can improve or impairs endothelial function is still controversial. Some studies have shown that mild to moderate alcohol consumption is associated with improved endothelial function [43–45]. Meanwhile, several studies suggest that mild to moderate alcohol consumption does not enhance endothelial function, while heavy drinking negatively impacts endothelial function [46–49]. However, a few studies have shown that even light alcohol intake can impair endothelial function [50, 51]. Second, ethanol can interfere with cholesterol synthesis in mice [52], some studies suggest that alcohol consumption may impact endothelial function via increased high-density lipoprotein or decreased low-density lipoprotein [50, 53–55], however, the results of the effects of alcohol consumption on the lipids in the blood are not consistent [56–58], a large of studies suggested that mild alcohol consumption increased high-density lipoprotein or decreased low-density lipoprotein [59–62], while some studies had not found mild alcohol consumption correlated with high-density lipoprotein or low-density lipoprotein [63–65]. Third, prior studies had suggested that ethanol may promote atherosclerosis, but polyphenol antioxidants in fermented beverages had been shown to reduce atherosclerosis in a dose-dependent manner [66–68]. Fourth, differences in methodology between studies may lead to different even opposite results, such as different definition and classification of individual’s alcohol exposure, the time and type of alcohol consumption, the study population and sample sizes of studies [69]. For example the accuracy of respondents in remembering how much they drink is more subject, and few people maintain a consistent level or style of drinking throughout their lives [70]. Fifth, to the best of our knowledge there are no long-term and multicenter randomized controlled trials that have examined whether alcohol consumption reduces arteriosclerosis, so the influence of uncontrolled or unknown confounding on results cannot be completely excluded, such as lifestyle behaviors, light-to-moderate drinkers have better lifestyle behaviors compared with non-drinking [71]. The positive impacts of consuming mild alcohol on arteriosclerosis in some studies may stem from more healthier lifestyles [72].

### Study limitations

There are several limitations of our study. First, the study subjects were acute ischemic stroke patients only from southwest Sichuan province; therefore, the findings are limited in their generalizability. Second, alcohol consumption was calculated on the basis of self-reported data, which may be subject to misclassification bias. Third, a fewer female drinkers included in our study may not represent the effect of alcohol consumption on cervical and cerebral stenosis in women. Fourth, the severity and duration of smoking were not stratified in our analysis, there may be synergistic effect of smoking and alcohol consumption on atherosclerosis. In addition, populations with asymtomatic carotid and cerebral artery stenosis or occlusion were not included in the current study, so we may interpretate our results with cautions.

## Conclusions

Higher alcohol consumption may associate with higher risk and more severity of carotid and cerebrovascular stenosis.

## Data Availability

All relevant data are within the paper.
